# THE ROLE OF COMMUNITY ENGAGEMENT IN HURRICANE PREPAREDNESS AS PERCEIVED BY ASSISTED LIVING ADMINISTRATORS

**DOI:** 10.1093/geroni/igz038.1438

**Published:** 2019-11-08

**Authors:** Debra J Dobbs, Joseph June, Lindsay J Peterson, David Dosa, Dylan Jester, Kathyrn Hyer

**Affiliations:** 1 University of South Florida, Tampa, Florida, United States; 2 Brown University, Providence, Rhode Island, United States

## Abstract

The importance of communities in disasters has been well established since Hurricane Katrina. Smit and Wandel’s bottom-up approach to assess risks during a disaster involves community stakeholders. Administrators of assisted living (AL) environments increasingly have to assess the risks of hurricane evacuation for vulnerable older adults. The current study examines intersections between social networks, communication and preparedness during a hurricane for AL administrators. We conducted focus groups and interviews with AL administrators (N=60) in Florida about communication patterns with community associations, emergency management officials, AL staff, residents and their families during Hurricane Irma (2017) and about their perceptions of preparedness. A content analysis approach was used. Atlas.ti v7 was used for initial and axial coding. Co-occurrences were found among communication and preparedness themes. Some prevalent themes included “social capital”, “high versus low tech strategies” and “leadership effectiveness”. Themes intersected with individual administrator and AL organizational characteristics.

